# Supplementary material for: Whole genome sequencing of Herpes Simplex Virus 1 directly from human cerebrospinal fluid reveals selective constraints in neurotropic viruses
Source: Virus Evol. 2020 Feb 20;6(1):veaa012. doi: 10.1093/ve/veaa012 (PMC7031915; doi:10.1093/ve/veaa012)
Supplement: veaa012_Supplementary_Materials [file veaa012_supplementary_materials.pdf]

Supplementary Table 1

List of non-synonymous mutations observed in the consensus sequence of sample HSV1-SWAB7 with respect to the reference strain 17 genome sequence (NCBI RefSeq accession NC_001806.2).

Gene	Genome pos.	CDS pos.	Prot. Pos.	Ref codon	Ref. AA	sample codon	sample AA	unique among CSF/SWAB samples?	reference allele occurs as minority var. at freq.
UL8	19420	1057	353	GCC	A	GTC	V		
UL8	18644	1833	612	TGC	C	GGC	G		
UL9	NA								
UL30	62903	96	33	AGC	S	GGC	G		
UL30	64080	1273	425	AAC	N	ACC	T	unique	0.084
UL30	66101	3294	1099	GCC	A	ACC	T		0.147
UL30	66144	3337	1113	TCC	S	TGC	C		0.223
UL30	66177	3370	1124	CCT	P	CAT	H		0.407
UL30	66197	3390	1131	GCG	A	TCG	S	unique	0.181
UL30	66428	3621	1208	ACC	T	GCC	A		
UL5	14933	199	67	CAT	H	CGT	R		
UL5	14519	613	205	TTG	L	TCG	S		
UL5	14033	1099	367	GAG	E	GCG	A	unique	
UL42	94241	1129	377	TTG	L	TCG	S		
UL52	109680	631	211	GTG	V	GCG	A		
UL52	110589	1540	514	GGC	G	GAC	D		
UL52	110591	1542	515	CCC	P	ACC	T		
UL52	110735	1686	563	CGC	R	TGC	C	unique	
UL52	111221	2172	725	GAT	D	AAT	N	unique	
UL52	111282	2233	745	GAA	E	GCA	A	unique	
UL29	61925	129	44	TCC	S	GCC	A		
UL29	61012	1042	348	TTC	F	TGC	C		

Supplementary Figures:

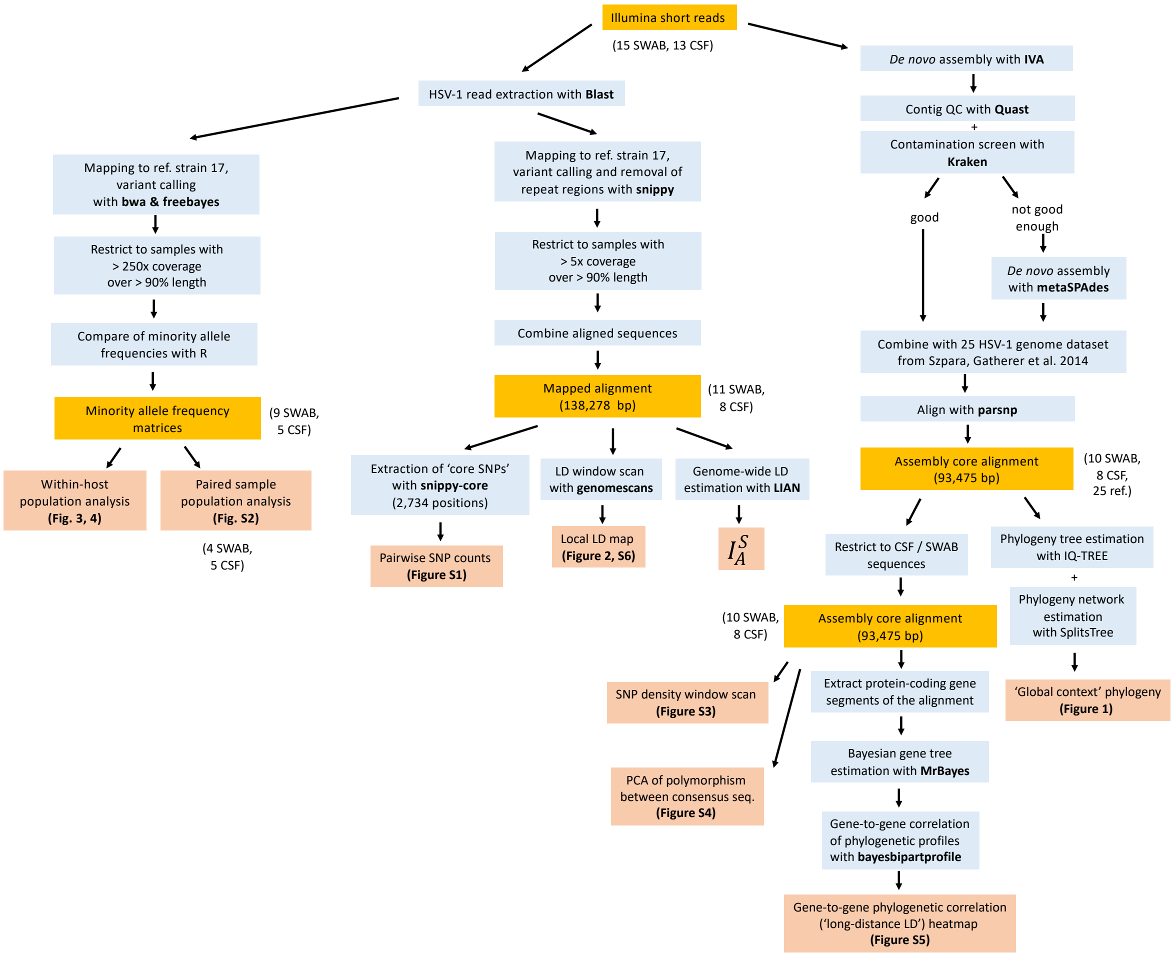

Supplementary Figure 1: Workflow of genomic analyses

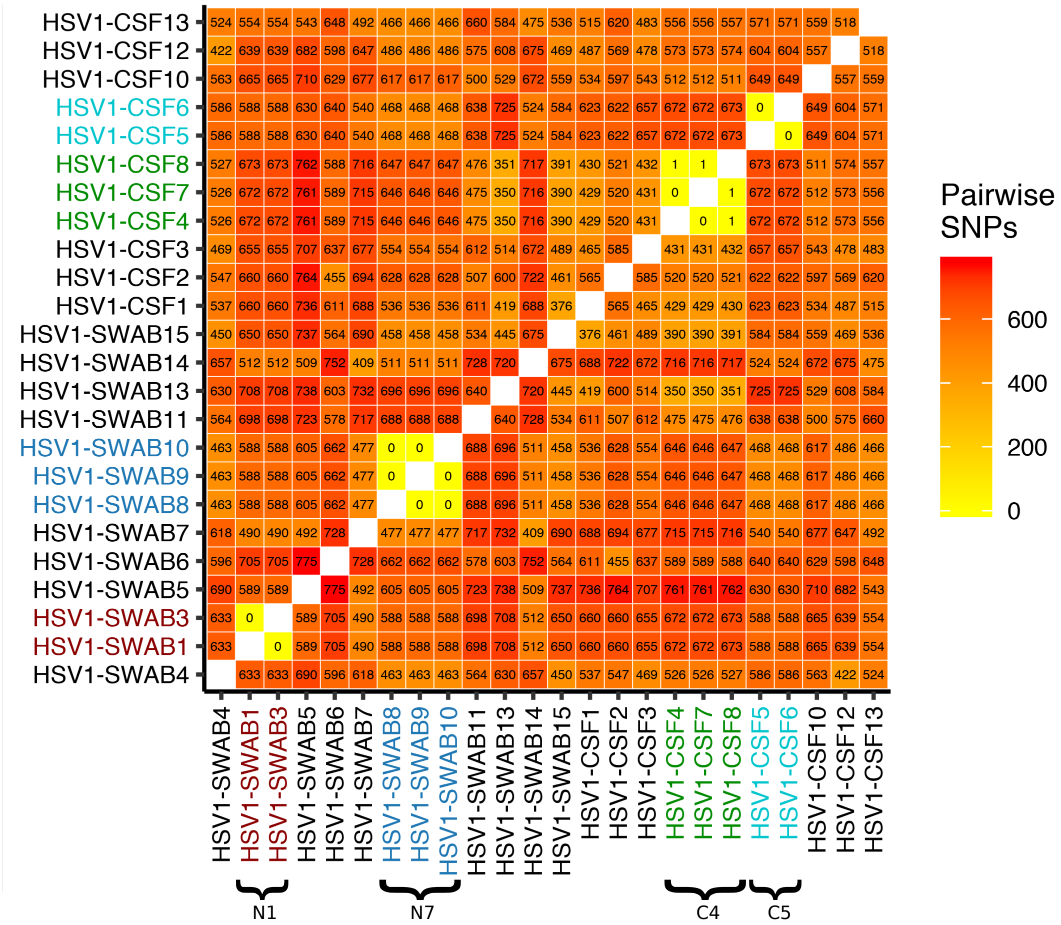

Supplementary Figure 2. Pairwise SNP differences between unrelated samples and samples taken from the same patient.

Unrelated samples are separated by >350 SNPs, while related samples have virtually identical consensus sequence (≤1 SNP) within the same patient. Samples from the same patient are indicated with brackets.

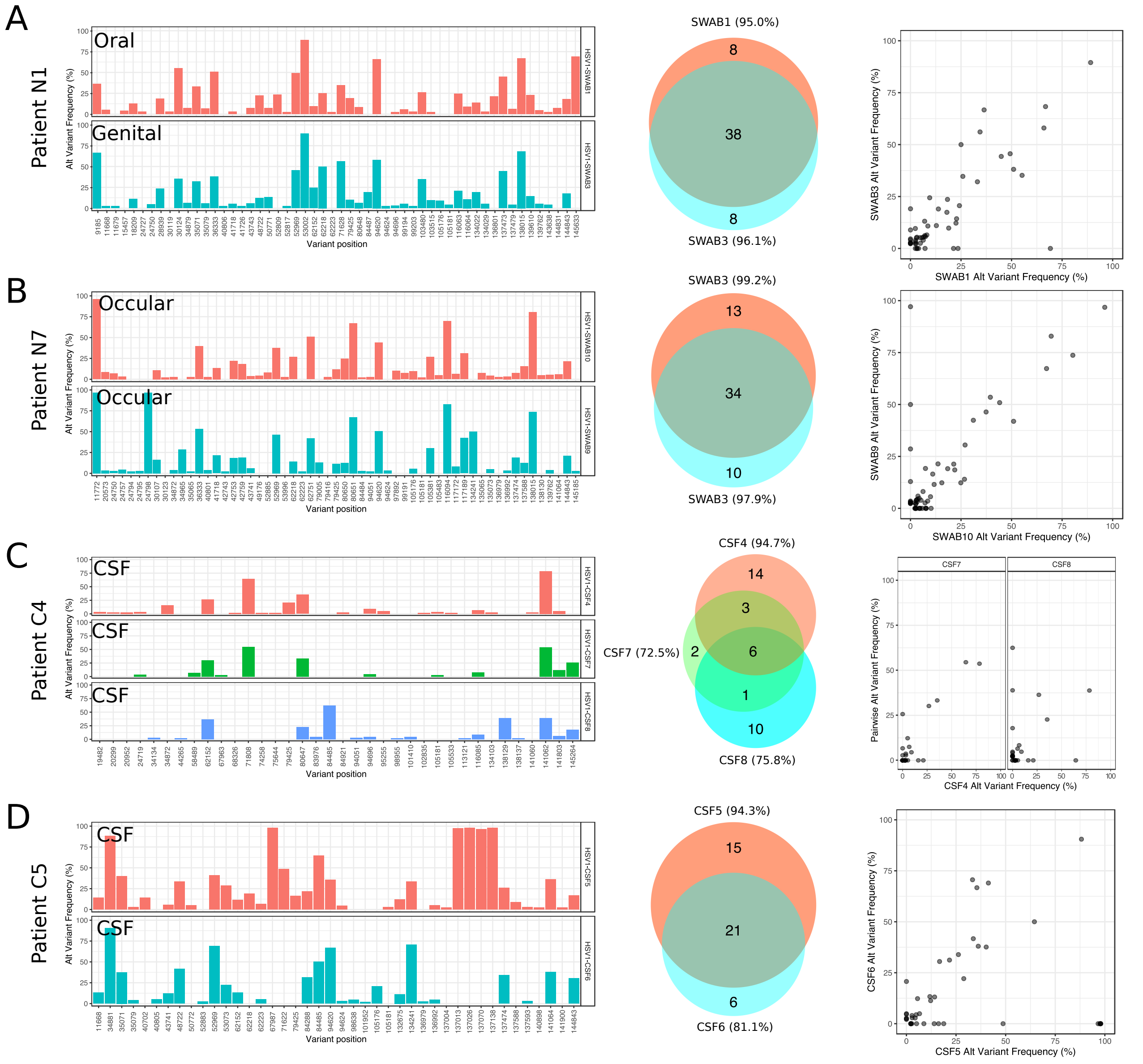

Supplementary Figure 3. Replication of minor variant allele profiles between paired samples from same patient.

Barplots show alternate (non-reference) allele frequencies by position. Venn diagrams indicate common minor variant positions; percentage coverage of genomes (for positions with > 250 reads) is indicated in parentheses. Scatter plots show frequencies of common variants in pairwise comparison. A. Patient N1, multiple sites. B. Patient N7, biological replicates from the same site. C. patient C4, technical replicates (same sample). D. Patient C5, biological replicates from the same site.

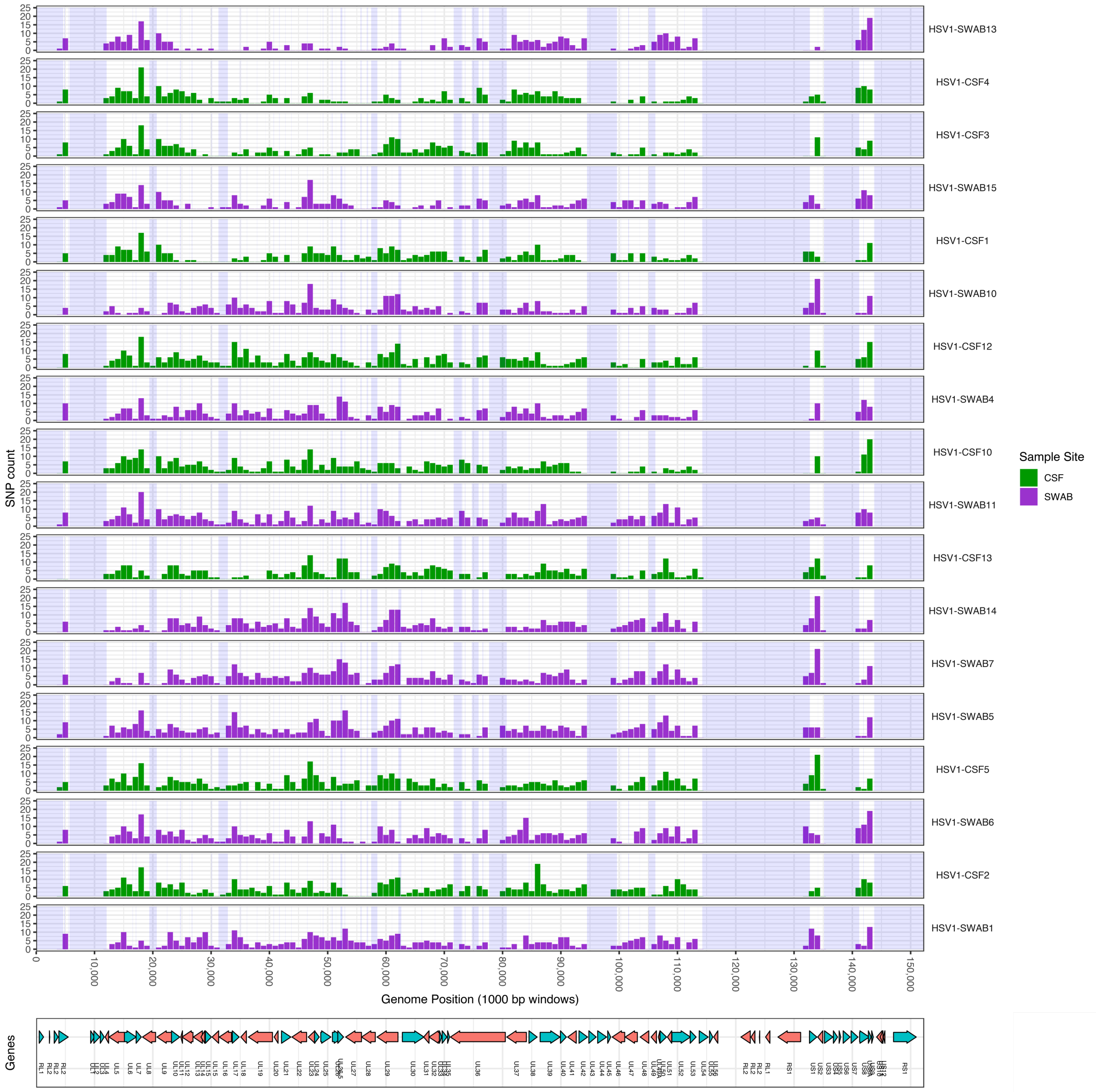

Supplementary Figure 4. Genome-wide SNP density per sample.

SNP density was plotted in sliding windows (500bp) along the genome in all samples from this study using the assembly core alignment. SNPs are broadly distributed along the genome, but increased diversity was observed in the US region. Gaps in the core-genome alignment are indicated with blue shade.

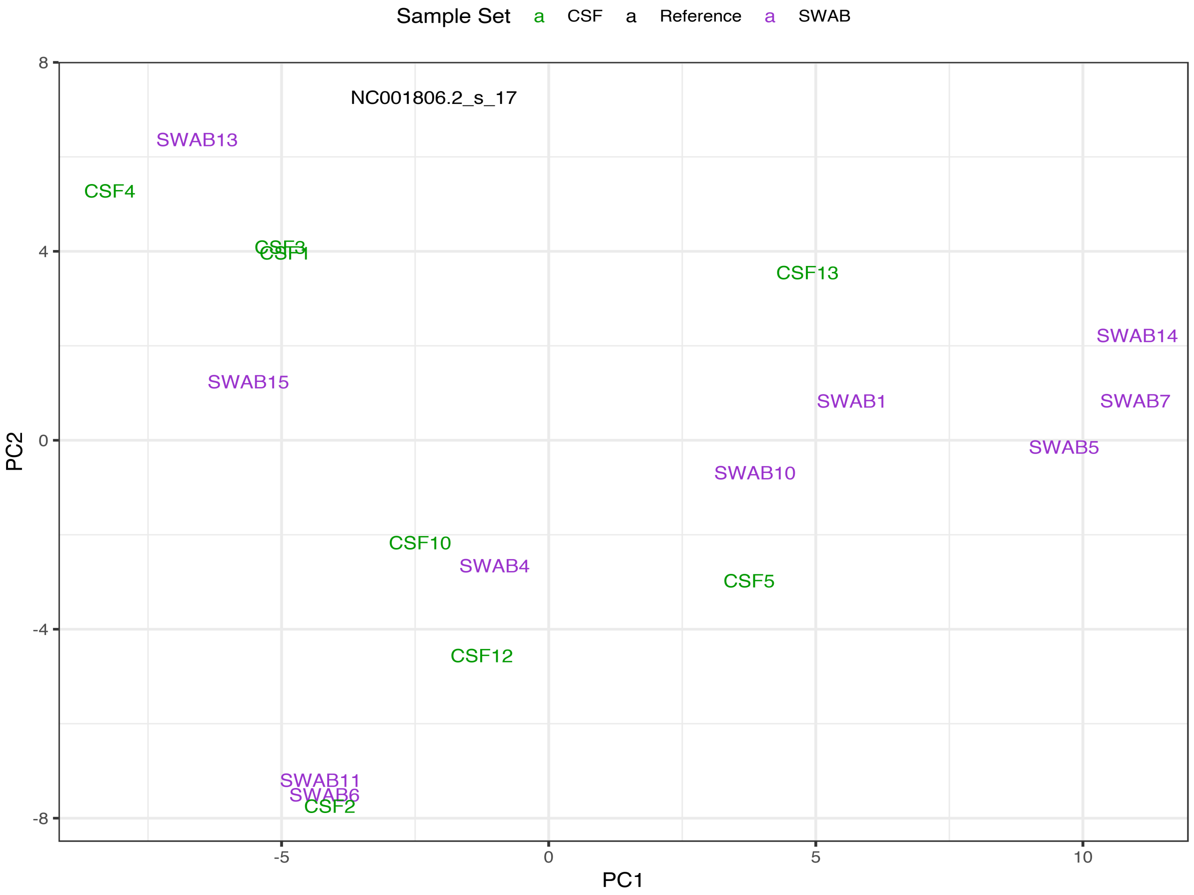

Supplementary Fig 5. PCA of covariance of polymorphisms between consensus sequences shows absence of strong population structure.

Samples coloured by sample category: green: CSF; purple: SWAB; black: reference.

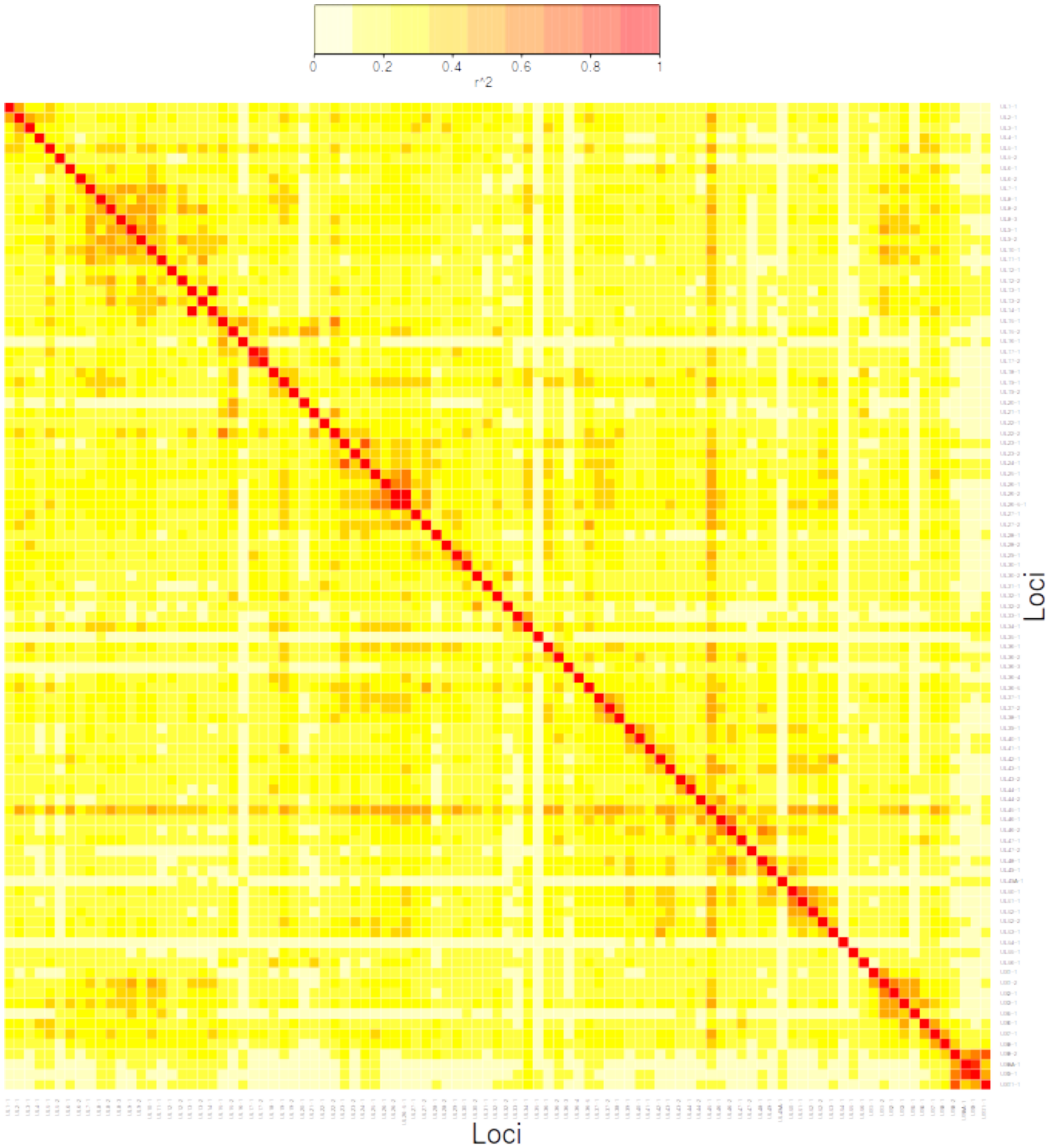

Supplementary Figure 6. Gene-to-gene correlation of phylogenetic profiles of population structure.

The phylogenetic relationships between consensus genomes were dissected for each gene locus using the bayesbipartprofile tool (https://github.com/flass/genomescans). Phylogenetic compatibility of tree splits with each gene alignment was computed for all splits observed in at least one Bayesian gene tree sample at a minimum frequency of 0.1 and separating clades of at least 3 sequences. The gene-to-split compatibility scores were then used to compute correlation (r^2) between gene pairs, displayed as a heatmap; genes are ordered following their position in the genome. Genes physically clustered together show significant correlation of phylogenetic structure (i.e. related history of descent), but not those located far apart in the genome. Elevated compatibility of UL45 with most other genes indicates limited phylogenetic information available in this gene's alignment.

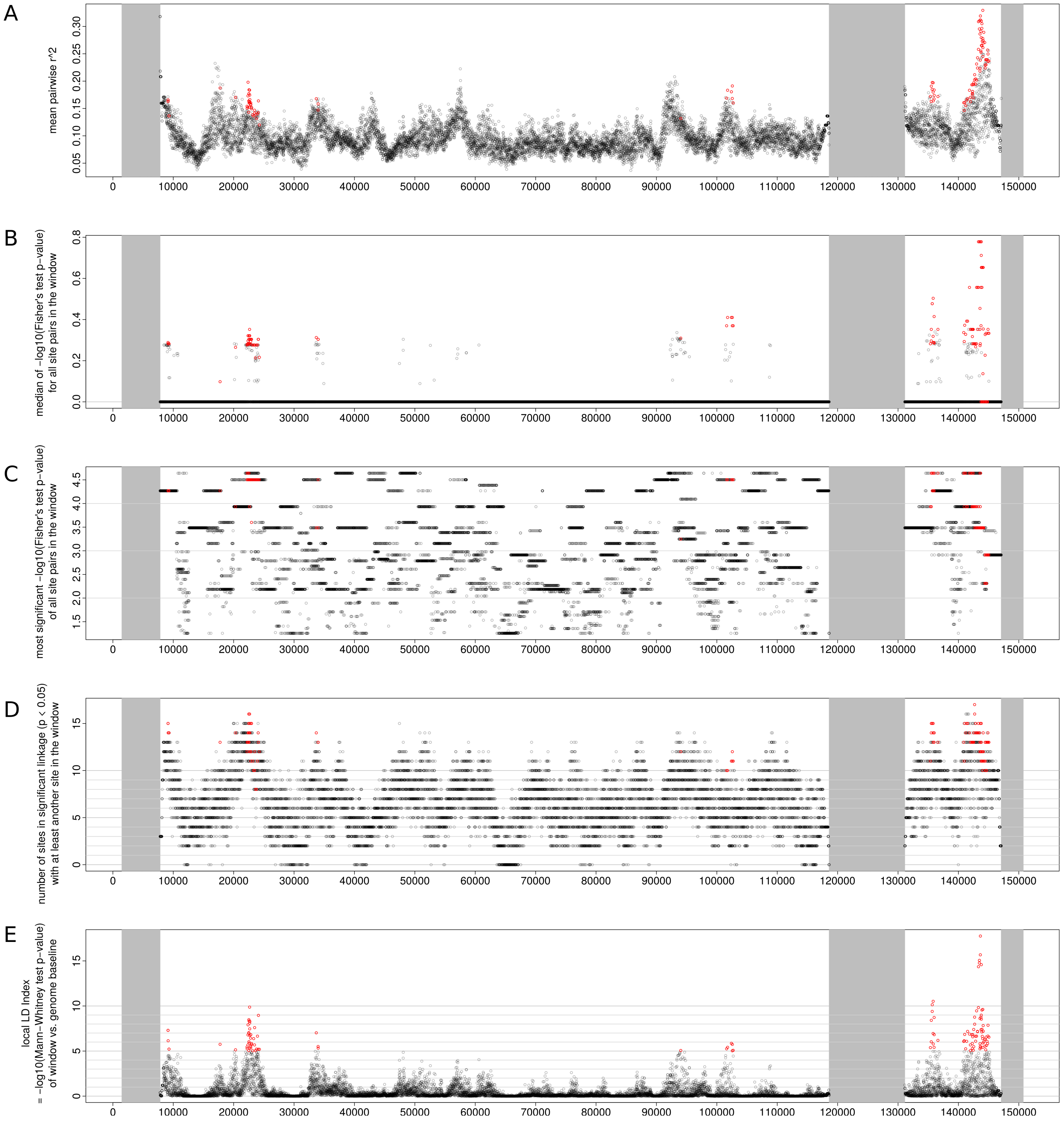

Supplementary Figure 7. Comparison of local LD metrics in genome-wide scan of 18 CSF and SWAB HSV-1 genomes.

A sliding window scan was conducted over the mapped alignment of 18 HSV-1 consensus genomes (3000bp windows, 10bp step; SNPs were subsampled so each window contains exactly 20 bi-allelic SNPs), and various metrics of within-window LD were reported, each aggregating in a different simpler LD metrics (r^2 or Fisher's exact test p-value) measured for each of the 190 pairs site present in a 20-site window): A) mean r^2 as in Figure 2A; B) median Fisher p-value (-$\log_{10}$ transformed); C) most significant Fisher p-value (-$\log_{10}$ transformed); D) number of sites in significant linkage (Fisher p-value < 0.05) with at least another site within the window; E) local LD index (LDI), which is the -$\log_{10}$ transformed p-value of a one-sided Mann-Whitney-Wilcoxon test comparing the set of Fisher test p-values within the window to the distribution of values observed in all windows across the genome. Dots in red indicate the windows with LDI > 5, reported as significant local LD peaks in Figure 2A.

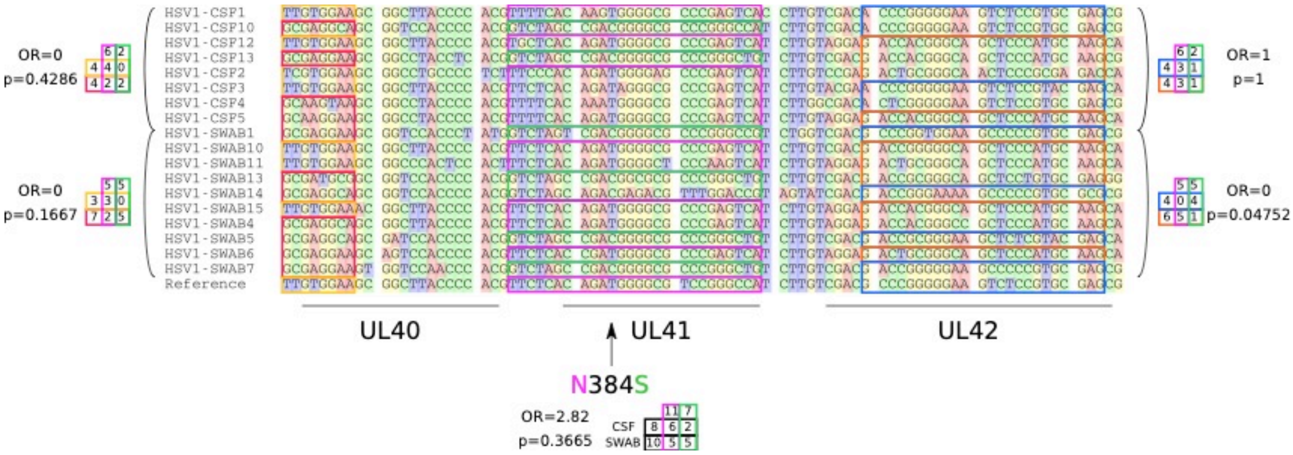

Supplementary Figure 8. Haplotype structure of UL40/41/42 locus.

Mapped alignment of consensus genomes from eight CSF and ten SWAB samples, reduced to the bi-allelic nucleotide sites occurring within the coordinates 89,900 to 94,600 (relative to reference strain 17), i.e. the region spanning the genes UL40, UL41 and UL42.

Haplotypes, i.e. stretches of sites that are phylogenetically congruent, are framed and colour-coded according to a binary grouping per gene locus. Sites without framing were deemed subject to too much recombination to be classified into haplotypes. Agreement of haplotype structures between gene loci is indicated separately for the CSF (top) and SWAB (bottom) groups for UL40 vs. UL41 (left) and UL41 vs. UL42 (right), respectively. Results of Fisher's exact tests for each group/comparison show that population structure is stronger across these loci within the SWAB group than within the CSF group.

The non-synonymous site (genome position 91,486) leading to an asparagine to serine change (N384S) in UL41 protein product Vhs is indicated as it was previously reported to be associated with an increased virulence in a mouse model of ocular infection (Kolb et al. 2016).
